# HCC-derived EGFR mutants are functioning, EGF-dependent, and erlotinib-resistant

**DOI:** 10.1186/s13578-020-00407-1

**Published:** 2020-03-16

**Authors:** Natthaporn Sueangoen, Anchalee Tantiwetrueangdet, Ravat Panvichian

**Affiliations:** 1 Research Center, Faculty of Medicine, Ramathibodi Hospital, Mahidol University, Bangkok, Thailand; 2 Department of Internal Medicine, Division of Medical Oncology, Faculty of Medicine, Ramathibodi Hospital, Mahidol University, Rama 6 Road, Ratchathewi, Bangkok, 10400 Thailand

**Keywords:** Apoptosis, Autophagy, Erlotinib-resistant, HCC-derived EGFR mutants, EGFR phosphorylation

## Abstract

**Background:**

Epidermal growth factor receptor (EGFR) has emerged as an important therapeutic target. Overexpression of EGFR is frequently observed in hepatocellular carcinoma (HCC) and EGFR activation has been proven to be a potential determinant of primary resistance of HCC cells to sorafenib. In our previous study, we found 13 missense mutations in EGFR exon 19–23 from hepatocellular carcinoma (HCC) tissues, but the functions of these mutations have not been determined. This study aims to determine the kinase activity and sensitivity to erlotinib, a 1st-generation EGFR-tyrosine kinase inhibitor (TKI), of seven HCC-derived mutants (K757E, N808S, R831C, V897A, P937L, T940A, and M947T).

**Results:**

Using transduction of pBabe-puro retroviral vector with or without EGFR, we constructed and determined the function of EGFRs in NIH-3T3 cells stably harboring each of the seven mutants, as well as the erlotinib-sensitive L858R-mutant, the erlotinib-resistant T790M-mutant, and EGFR wild type (WT). Our results indicate that the seven mutants are functioning, EGF-dependent, EGFRs. Cells harboring six of the seven mutants could generate some level of EGFR phosphorylation in the absence of EGF, indicating some constitutive kinase activity, but all of the seven mutants remain primarily EGF-dependent. Our results demonstrate that erlotinib induces differential degree of apoptosis and autophagy among cells harboring different EGFRs: complete apoptosis and autophagy (cleavage of both caspase-3 and PARP, and marked LC3-II increment) in L858R-mutant; partial apoptosis and autophagy (only cleavage of caspase-3, and moderate LC3-II increment) in WT and HCC-derived mutants; and no apoptosis and minimal autophagy (no cleavage of caspase-3 and PARP, and minimal LC3-II increment) in T790M-mutant. The seven HCC-derived mutants are erlotinib-resistant, as treatment with erlotinib up to high concentration could only induce partial inhibition of EGFR phosphorylation, partial or no inhibition of AKT and ERK phosphorylation, and partial apoptosis and autophagy.

**Conclusion:**

The seven HCC-derived EGFR mutants in this study are functioning, EGF-dependent, and erlotinib-resistant. Erlotinib induces differential degree of apoptosis and autophagy among cells harboring different EGFRs. The degree of inhibition of EGFR phosphorylation by erlotinib is the determining factor for the degree of apoptosis and autophagy amongst cells harboring EGFR mutants. This study paves the way for further investigation into the sensitivity of these HCC-derived mutants to the 3rd-generation irreversible EGFR-TKI, osimertinib.

## Introduction

In 2018, hepatocellular carcinoma (HCC) is the fourth leading cause of cancer death worldwide [1]. About 80–90% of HCC is associated with cirrhosis developed from chronic infection with hepatitis B virus (HBV) or hepatitis C virus (HCV) [2]. Being diagnosed at the advanced stage, the majority of patients with HCC are not the candidates for potentially curative therapies such as surgical resection and transplantation [3]. Sorafenib, a multi-kinase inhibitor introduced in 2007, was the first systemic agent to demonstrate a significant improvement in overall survival of advanced HCC patients in two phase III trials and has become the standard first-line treatment for unresectable HCC since then [4, 5]. Lenvatinib has recently been proven to be non-inferior to sorafenib in overall survival in untreated advanced hepatocellular carcinoma [6]. Besides, two other kinase inhibitors (regorafenib, cabozantinib) have recently been shown to prolong survival of patients with advanced HCC patients progressing on/after sorafenib treatment [7, 8]. However, primary and acquired resistance to the multi-kinase inhibitors is observed in these patients.

Epidermal growth factor receptor (EGFR) is a member of the family of receptor tyrosine kinases (RTKs). Upon binding with its ligands in the epidermal growth factor (EGF) family, EGFR undergoes dimerization and formation of the asymmetric (activator-receiver) kinase dimer, leading to the active conformation of tyrosine-kinase-domain (TKD) in the receiver subunit which then phosphorylates key tyrosine residues in the c-terminal tail of EGFR, which consequently function as specific docking sites for cytoplasmic proteins containing phosphotyrosine-binding domains, resulting in assembly and activation of downstream signaling molecules [9, 10]. Aberrations in EGFR activation through EGFR gene amplification, mutations, and/or overexpression have been detected in various cancers and causally linked to poor prognosis of the patients [11, 12]. EGFR has emerged as an important therapeutic target for cancer treatment. The inhibitors targeting TKD of EGFR have been approved in non-small cell lung cancer; however, these EGFR tyrosine kinase inhibitors (TKIs) are especially more active in constitutively active mutant EGFRs, most notably exon19-deletion and exon 21-L858R, than in wild-type (WT) EGFR [13–17].

Overexpression of EGFR is frequently observed in HCC [18], suggesting that EGFR might play an important role in HCC pathogenesis and treatment. Furthermore, EGFR activation has been proven to be a potential determinant of primary resistance of hepatocellular carcinoma cells to sorafenib [19, 20]. However, EGFR overexpression in HCC does not correlate with the gains of the EGFR gene copy number. Besides, EGFR gene amplification does not occur in HCC [21]. In addition, the absence of somatic missense mutations in EGFR exon 18–21 from HCC tissues has been reported by 2 groups of investigators using direct sequencing [22, 23]. Nevertheless, in our previous study using PCR cloning and sequencing, we found 13 novel missense mutations in EGFR exon 19–23 from HCC tissues [24], but the biological functions of these missense mutations have not yet been determined.

Therefore, we inquire whether these HCC-derived EGFR mutants can generate EGFR tyrosine phosphorylation in the absence of EGF, an indicator of constitutively active EGFR, and whether they are sensitive to erlotinib, an active 1st-generation EGFR-TKI. To answer these questions, we did experiments in mouse fibroblast NIH-3T3 cells stably harboring each of seven HCC-derived EGFR missense mutants (K757E, N808S, R831C, V897A, P937L, T940A, and M947T), as well as the erlotinib-sensitive L858R-mutant, the erlotinib-resistant T790M-mutant, EGFR wild type (WT), and vector without EGFR. These cells were constructed by transduction of pBabe-puro retroviral vector with or without EGFR into NIH-3T3 cells, which do not express endogenous EGFR. The EGFR kinase activity in these cells, when treated with or without EGF, was evaluated by determining their EGFR phosphorylation at Tyr869 (Y869) located in the activation loop and Tyr1092 (Y1092) located in the c-terminal tail of EGFR. The phosphorylation of AKT and ERK was used as indicators of the activation of downstream signaling molecules of EGFR. We determined cell proliferation in these cells up to 5 days, as an indicator of the global effect of EGFR activation. Then, we correlated their EGFR, AKT and ERK phosphorylation status with their cell proliferation and sensitivity to erlotinib, as determined by MTT assay. We also investigated their erlotinib-sensitivity with the induction of erlotinib-induced apoptosis and/or autophagy.

## Materials and methods

### Structures of HCC-derived EGFR mutants

The structures of HCC-derived EGFR mutants in this study were generated by the modification of crystal structures, deposited in the Protein Data Bank (PDB), of wild-type EGFR in complex with ATP analog-peptide conjugate (PDB ID: 2GS6) [25], and with erlotinib (PDB ID: 1M17) [26]. The modified EGFR structures were generated and drawn using the PyMOL Molecular Graphics System (Version 2.3.2) (Schrodinger, New York, NY, USA).

### Cell culture and reagents

NIH-3T3 and HEK293T cells were obtained from the American Type Culture Collection (ATCC). NIH-3T3 cell line is well known for having no endogenous EGFR expression, as determined by immunoblotting [27, 28]. These were maintained in Dulbecco's modified Eagle's medium (DMEM) (GIBCO, NY, USA) supplemented with 10% fetal bovine serum (FBS)(GIBCO, NY, USA), 100 U/mL Penicillin, 100 µg/mL Streptomycin, 0.25 µg/mL Amphotericin B (GIBCO, NY, USA) and cultivated at 37 °C in humidified atmosphere of 5% CO_2_. Cells were sub-cultured when cells reached 80–90% confluent with 0.25% trypsin solution.

### Construction of plasmids with HCC-derived EGFR mutants & retroviral transduction to NIH-3T3 cells.

Seven missense mutations in the kinase domain of EGFR including K757E, N808S, R831C, V897A, P937L, T940A and M947T were generated individually from pBabe-puro/EGFR wild-type (WT) vector (Addgene, MA, USA) by QuikChange XL Site-Directed Mutagenesis Kit (Agilent, CA, USA) according to the manufacturer’s instructions with the following primers shown in Table 1. pBabe-puro/EGFR T790M (Addgene, MA, USA) and pBabe-puro/EGFR L858R (Addgene, MA, USA) were used as erlotinib-resistant mutant control and erlotinib-sensitive mutant control, respectively. The mutated DNA sequences in these pBabe-puro/EGFR mutants were then confirmed with sequencing by Macrogen Inc., South Korea (Additional file 1: Figure S1). NIH-3T3 cells stably harboring pBabe-puro (vector alone), or EGFR wild-type (WT) or EGFR mutants were generated individually by retroviral transduction via co-transfection of envelope and packaging plasmids (pCMV-VSV-G and pUMVC) with transfer plasmids (vector alone or EGFR WT or EGFR mutants) (Addgene, MA, USA) into HEK293T cells by Lipofectamine LTX with Plus™ Reagent (Invitrogen, CA, USA). Then, after 72 h of transfection, retrovirus in the medium was transduced into mouse fibroblast NIH-3T3 cells, which do not express endogenous EGFR, by exposure to Polybrene (Milipore, CA, USA) for 24 h. The stable clones were selected from transduced NIH-3T3 cells by treatment with 2 µg/ml Puromycin (Santa Cruz Biotechnology Inc., CA, USA) for one week and these drug-resistant transduced NIN-3T3 cells were expanded for further experiments. Three different clones of NIH-3T3 cells stably harboring EGFR WT, individual of EGFR mutants, and pBabe-puro (vector alone) were used for finding mean ± standard error of the mean (SEM) in the following experiments.

**Table 1:** List of EGFR primer sequences used in the study

EGFR mutation	Primer sequences
K757E	
Forward primer	5’CATCTCCGAAAGCCAACGAGGAAATCCTCGATGAAG3’
Reverse primer	5’CTTCATCGAGGATTTCCTCGTTGGCTTTCGGAGATG3’
N808S	
Forward primer	5’GGGAACACAAAGACAGTATTGGCTCCCAGTAC3’
Reverse primer	5’GTACTGGGAGCCAATAC TGTCTTTGTGTTCCC3’
R831C	
Forward primer	5’CTACTTGGAGGACTGTCGCTTGGTGCAC3’
Reverse primer	5’GTGCACCAAGCGACAGTCCTCCAAGTAG3’
V897A	
Forward primer	5’CACCAGAGTGATGCCTGGAGCTACG3’
Reverse primer	5’CGTAGCTCCAGGCATCACTCTGGTG3’
P937L	
Forward primer	5’CCTCCCTCAGCCACTCATATGTACCATCG3’
Reverse primer	5’CGATGGTACATATGAGTGGCTGAGGGAGG3’
T940A	
Forward primer	5’CTCAGCCACCCATATGTGCCATCGATGTCTACATG3’
Reverse primer	5’CATGTAGACATCGATGGCACATATGGGTGGCTGAG3’
M947T	
Forward primer	5’CGATGTCTACATGATCACGGTCAAGTGCTGGATG3’
Reverse primer	5’CATCCAGCACTTGACCGTGATCATGTAGACATCG3’

### Immunoblotting of EGFR, phosphorylated EGFR (pEGFR), downstream signaling molecules, and molecular markers of apoptosis and autophagy

NIH-3T3 cells stably harboring vector alone, or EGFR WT and mutants were starved with serum-free media for 24 h and then treated with or without 50 ng/ml EGF for 30 min. Protein extraction was performed using RIPA lysis and Extraction buffer (Pierce, IL, USA) with 1X protease inhibitor cocktail (Roche Diagnostics, IN, USA), 40 mM β-glycerophosphate, 50 mM NaF, 2 mM Na_3_VO_4_, 1 mM dithiothreitol (DTT) and measured the protein concentration by Pierce™ BCA Protein Kit (Thermo Scientific, IL, USA). Protein samples were separated on SDS-(Bio-Rad Laboratories) and transferred onto polyvinylidene difluoride (PVDF) Immobilon membranes (Millipore, Darmstadt, Germany). The membranes were blocked with 5% skim milk in Tris-buffered saline (TBS) buffer (10 mM Tris, 150 mM NaCl, pH 8.0) containing 0.1% Tween-20 for 1 h at room temperature and probed overnight at 4 °C with specific primary antibodies.

For detecting protein expression of EGFR and phosphorylated EGFR, the following primary antibodies were used: Anti-EGFR antibody (#MA5-13,269) (Invitrogen, IL, USA), Anti-phospho-EGFR specific antibodies for Tyr845 (#07,820) (Milipore, CA, USA) and Tyr1068 (#04,339) (Merck, MA, USA). Note that EGFR has two numbering systems. The first system applied by Ullrich et al. excludes the 24-residue signal peptide, yielding numbers that correspond to the mature protein [29]. The second system used in the Uniprot knowledge base includes the signal peptide, yielding numbers that correspond to the nascent protein. Although the use of the first system (mature protein numbering system) is established in the literature, it is more convenient to use the second system (nascent protein numbering system) when moving from DNA to RNA and then to protein [30]. Commercial antibodies, such as the Tyr845- and Tyr1068-specific anti-phospho-EGFR, use the first system. In the second system, which was used throughout this study, Tyr845 is Tyr869 (Y869), and Tyr1068 is Tyr1092 (Y1092), respectively.

For detecting the phosphorylation of downstream signaling molecules of EGFR (i.e., AKT and ERK), the following primary antibodies (Cell Signaling Technology, MA, USA) were used: AKT Antibody #9272, Phospho-AKT (Ser473) (193H12) Rabbit mAb #4058, p44/42 MAPK (ERK1/2) Antibody #9102, and Phospho-p44/42 MAPK (ERK1/2) (Thr202/Tyr204) (D13.14.4E) XP^®^ Rabbit mAb #4370.

For detecting erlotinib-induced apoptosis and autophagy, the following primary specific antibodies were used: Caspase-3 (#9665), Cleaved Caspase-3 (#9664), PARP (#9542), Cleaved PARP (#5625), and LC3-I/II (#12,741) (Cell Signaling Technology, MA, USA). β-Actin antibody (#MA5-15,739) (Thermo Fisher Scientific, IL, USA) was used for protein loading control.

Then, the membranes were incubated with secondary antibodies conjugated with horseradish peroxidase: anti-Rabbit IgG (#AP132P) or anti-Mouse IgG (#AP124P) (Milipore, CA, USA) for 1 h at room temperature. Protein bands were detected using Luminata™ Forte Western HRP Substrate (Merck, MA, USA), visualized on a LI-COR Odyssey IR imaging system and quantified band intensity using ImageJ program.

### MTT assay for cell proliferation and sensitivity to erlotinib

NIH-3T3 cells stably harboring vector alone, or EGFR WT and mutants (2 × 10^3^ cells) were plated into 96-well plates in triplicate and were allowed to adhere overnight. The cells were then treated with DMEM containing 4% FBS with or without 50 ng/ml EGF (Sigma) and replaced every 2 days. Cell proliferation was determined every day, for 5 days, by replacing culture media with 0.5 mg/ml MTT reagent (AppliChem, Darmstadt, Germany) and incubating at 37 °C for 4 h. The reagent was then removed and cell-crystals were dissolved in 150 µl DMSO and the absorbance was measured at 570 nM. Cell proliferation curves of these NIH-3T3 cells were based on the mean of the absorbance from triplicate wells, then means ± SEM from the three independent experiments were calculated and plotted as fold increase of cell proliferation compared to day 0, using GraphPad Prism 6 program.

To test sensitivity to erlotinib, 4 × 10^3^ NIH-3T3 cells stably harboring vector alone, EGFR WT and mutants were seeded into 96-well plates in triplicate overnight. These cells were then treated with erlotinib (Santa Cruz Biotechnology Inc., CA, USA) at various concentrations from 0.0001 to 5 µM for 72 h by replacing new media with erlotinib every 2 days. Viability of the cells treated with erlotinib, as an indicator of erlotinib sensitivity, was determined by MTT assay. Cell viability curves of cells treated with erlotinib were based on the mean of absorbance from triplicate wells, then means ± SEM from the three independent experiments were plotted as percentage of control without erlotinib at the end of 72 h, using GraphPad Prism 6 program.

### Statistical analysis

The data were presented as mean ± standard error of the mean (SEM) by one-way analysis of variance (one-way ANOVA) with LSD’s post-hoc test using Statistical Package for Social Science (SPSS) software version 17.0 (SPSS Inc., Chicago, IL, USA). Each assay was performed in triplicate. Data are considered significantly different when *P-*value is < 0.05.

## Results

### *H*CC-derived EGFR mutants are functioning, EGF-dependent, EGFRs

**Fig. 1:**
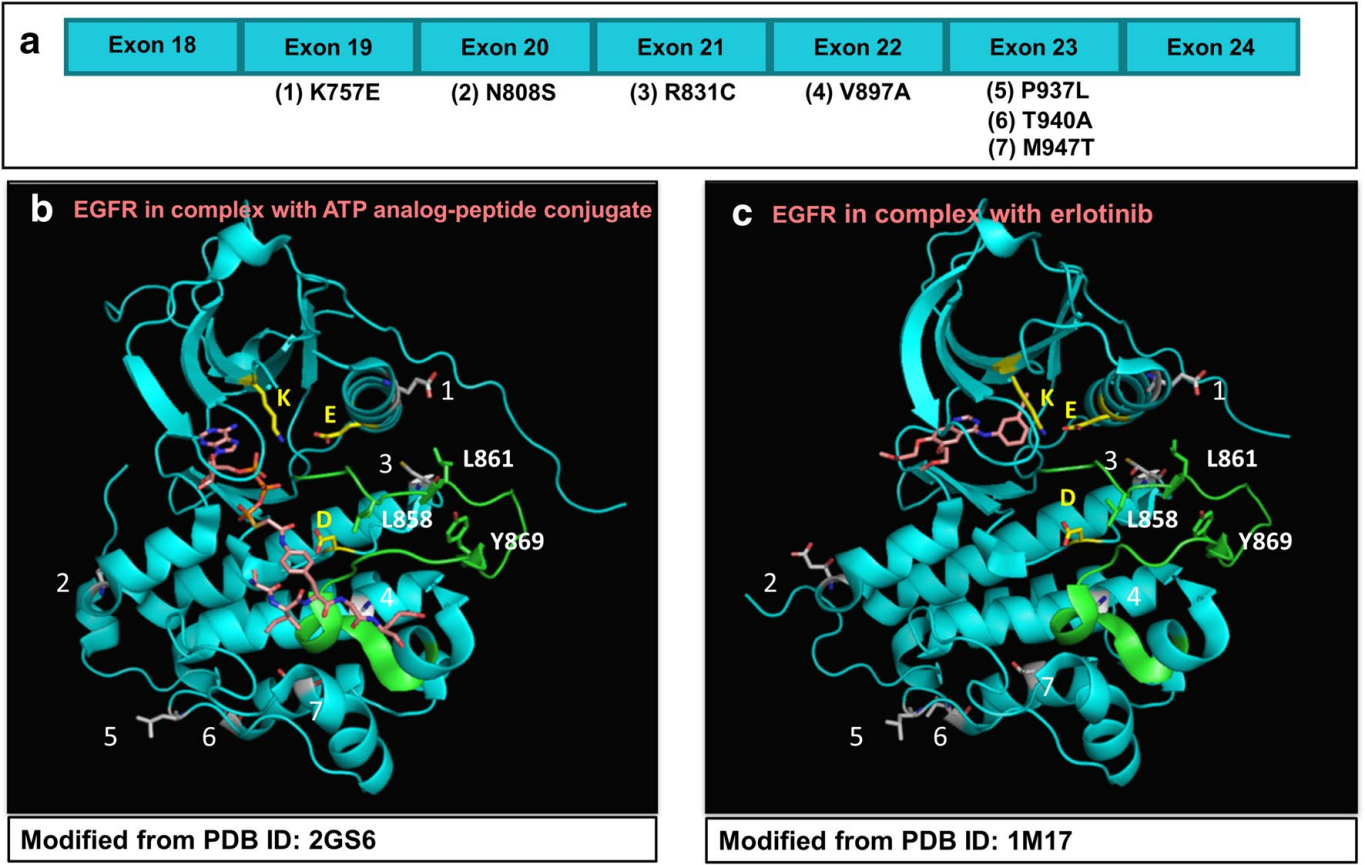
Structures of HCC-derived EGFR mutants, based on wild type EGFRs [25, 26]. Seven HCC-derived EGFR mutants arranged according to their codon position in EGFR exon 18–24, shown in graphic (**a**). Structures of these HCC-derived EGFR mutant residues in the active EGFR kinase domains are represented by modification of the wild type EGFR residues (PDB ID: 2GS6, and PDB ID: 1M17) [25, 26], with PyMOL Molecular Graphic System (Version 2.3.2) (Schrodinger, New York, NY, USA) (**b**, **c**, respectively). Overall, EGFR presented in cartoon with cyan color, whereas its conserved residues with ionic charges presented in sticks with carbon atom in yellow (K, K745; E, E762; D, D837), activation loop (855–884) in green and its important residues in sticks with carbon atom in green (L858, L861, Y869). HCC-derived EGFR mutant residues in this study presented in sticks with carbon atom in grey-white, numbering of the mutant residues as follows: 1, K757E; 2, N808S; 3, R831C; 4, V897A; 5, P937L; 6, T940A; 7, M947T. ATP analog-peptide conjugate and erlotinib presented in sticks with carbon atom in rose. In all sticks, nitrogen atom presented in blue, oxygen atom in red, and phosphorus atom in orange

The crystal structures of HCC-derived EGFR mutants in this study were generated and drawn by PyMOL Molecular Graphics System (Version 2.3.2) based on information from the crystal structures of wild-type EGFR in complex with ATP analog-peptide conjugate (PDB ID: 2GS6) [25], and with erlotinib (PDB ID: 1M17) [26]. The structures of HCC-derived EGFR mutants are shown in Fig. 1.

**Fig. 2:**
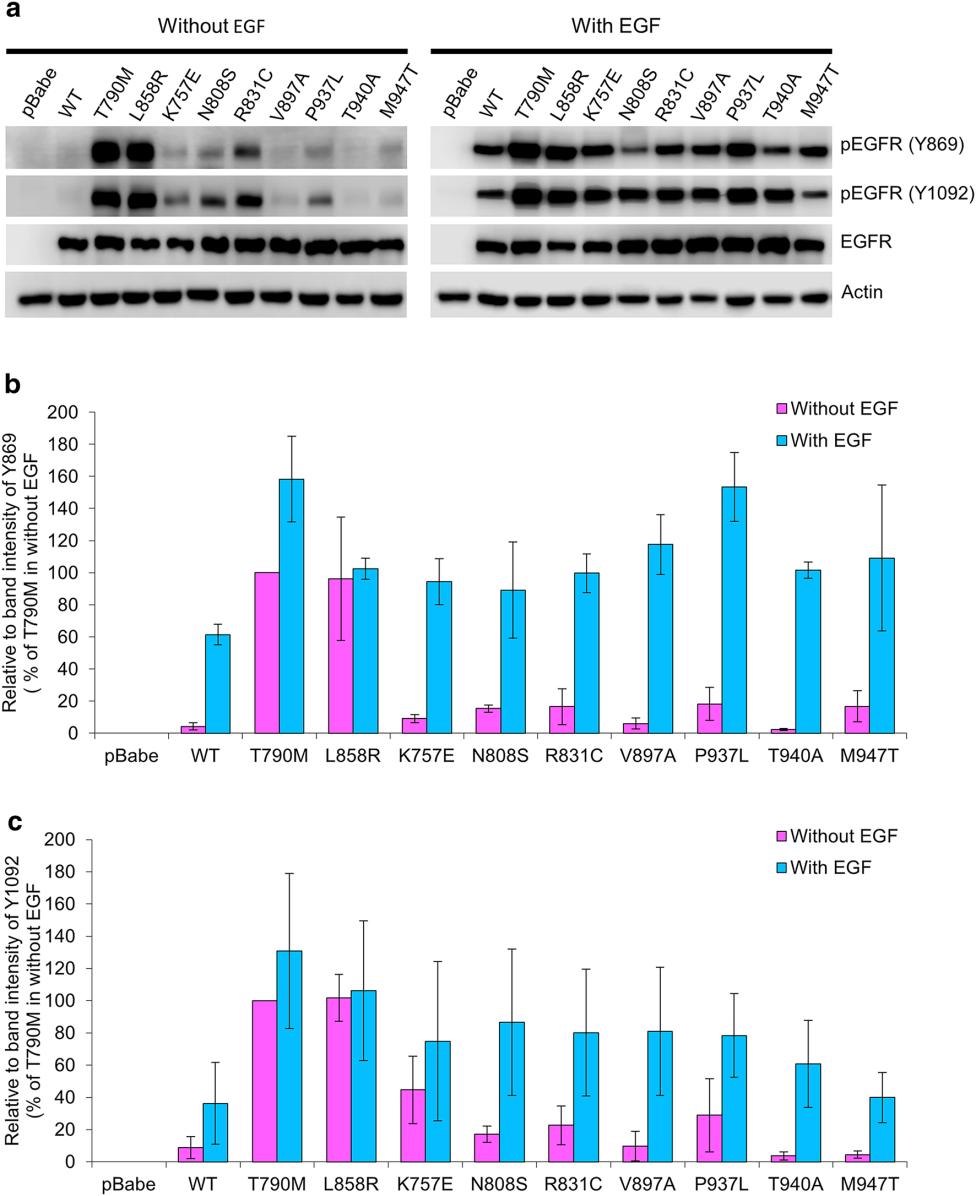
Protein Expression of EGFR and phosphorylated EGFR at Tyr869, Tyr1092. NIH-3T3 cells harboring pBabe-puro, EGFR WT and mutants were starved with serum-free media for 24 h and were then treated with or without 50 ng/ml EGF for 30 min. Protein extracts of these cells were analyzed by immunoblotting for EGFR and phosphorylated EGFR at Tyr869 and Tyr1092 [pEGFR (Y869), pEGFR (Y1092)], as described in the “Materials and methods” section, β-actin used as a loading control (**a**). Graphic presentations of relative band intensities of Tyr869 and Tyr1092 of each sample are shown as mean ± SEM (**b**, **c** respectively)

The protein expression of EGFR and phosphorylated EGFR (pEGFR) at Tyr869 (Y869), and Tyr1092 (Y1092) was detected using immunoblotting **(**Fig. 2). In NIH-3T3 cells harboring vector alone (pBabe-puro), the expression of EGFR and pEGFRs was not detectable at all, confirming no endogenous EGFR expression in NIH-3T3 cells. In all NIH-3T3 cells harboring EGFR wild-type (WT) and mutants, a similar level of EGFR expression was detected, using β-Actin as a loading control (Fig. 2a).

Without EGF treatment, NIH-3T3 cells harboring EGFR WT and EGFR-T940A displayed an almost undetectable level of pEGFR at Y869 and Y1092. In contrast, without EGF treatment, cells harboring T790M and L858R similarly displayed a high level of pEGFR at Y869 and Y1092, indicating their constitutively active TKD (Fig. 2). Besides, without EGF treatment, cells harboring six HCC-derived EGFR mutants (K757E, N808S, R831C, V897A, P937L, and M947T) also displayed some level of pEGFR at Y869 in the range of 6–18%, and at Y1092 in the range of 5–45%, of the level in cells harboring T790M without EGF treatment, which was calibrated as 100%; furthermore, four of them (K757E, N808S, R831C, and P937L) clearly displayed pEGFR at both Y869 and Y1092, indicating some constitutive kinase activity (Fig. 2).

With EGF treatment, NIH-3T3 cells harboring EGFR WT, T790M, L858R, and all seven HCC-derived EGFR mutants displayed increased levels of pEGFR at Y869 and Y1092, as compared with their levels without EGF (Fig. 2). With EGF treatment, cells harboring HCC-derived EGFR mutants displayed increased levels of pEGFR at Y869 in the range of 89–153%, and at Y1092 in the range of 40–86%, of the level in cells harboring T790M without EGF treatment, which was calibrated as 100%. Notably, the increments of pEGFR levels in cells harboring L858R treated with EGF were the least of all cells studied, and less than those of T790M and WT, indicating that the constitutive kinase activity of L858R-mutant has almost reached its full activity by itself without EGF treatment. However, the pEGFR levels in cells harboring HCC-derived EGFR mutants treated with EGF were much higher than their baseline without EGF, indicating that these HCC-derived EGFR mutants still response to EGF treatment and remain primarily EGF-dependent.

**Fig. 3:**
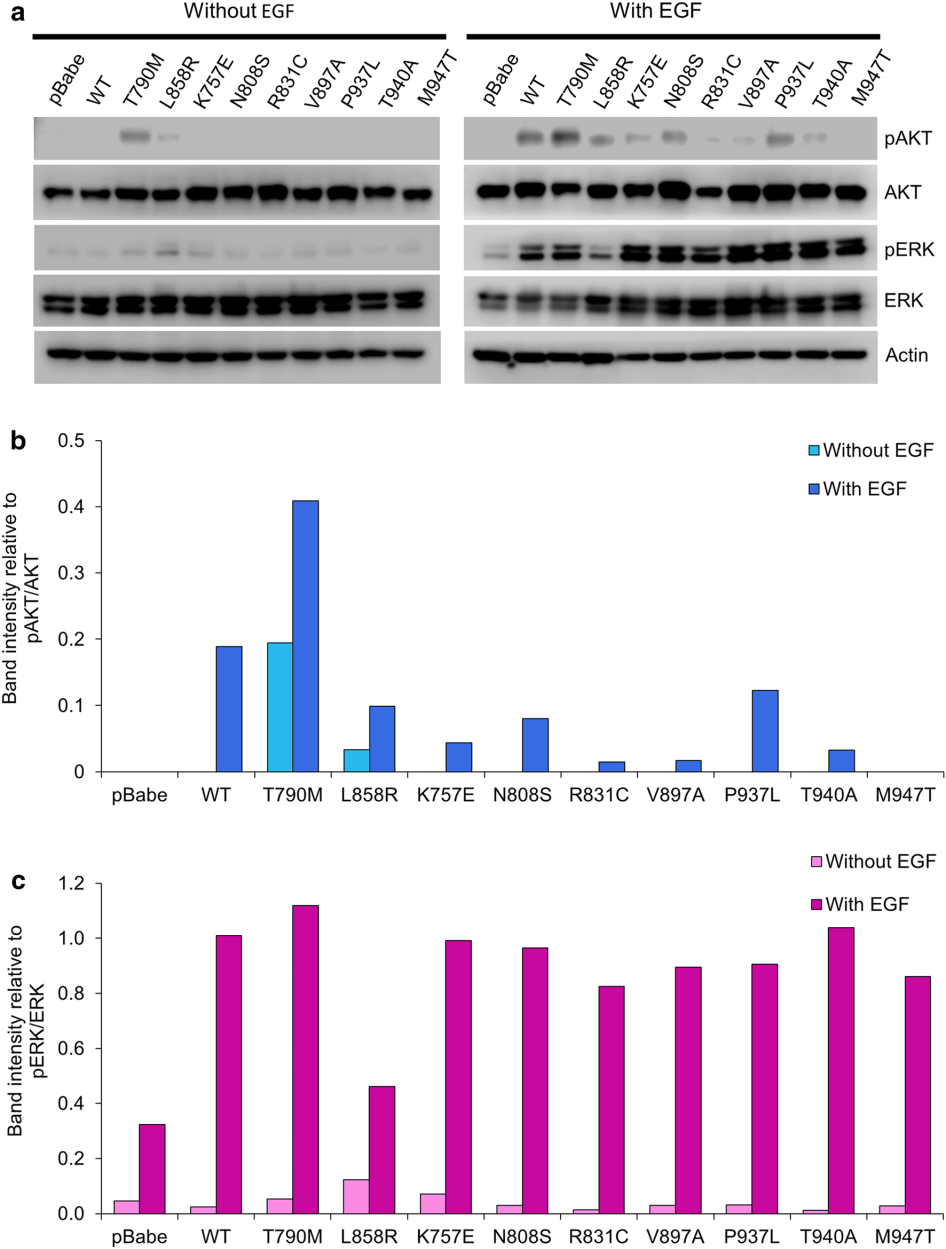
Protein Expression of downstream signaling molecules (AKT, phosphorylated AKT, ERK, and phosphorylated ERK). NIH-3T3 cells harboring pBabe-puro, EGFR WT and mutants were starved with serum-free media for 24 h and were then treated with or without 50 ng/ml EGF for 30 min. Protein extracts of these cells were analyzed by immunoblotting for downstream signaling molecules, as described in the “Materials and methods” section, β-actin used as a loading control (**a**). Graphic presentations of relative band intensities of these downstream signaling molecules of each sample are shown as mean ± SEM (**b, c**)

The protein expression of AKT, phosphorylated AKT (pAKT), ERK, phosphorylated ERK (pERK) was detected using immunoblotting (Fig. 3). In all NIH-3T3 cells harboring EGFR wild-type (WT) and mutants, comparable levels of AKT and ERK expression were detected, using β-Actin as a loading control (Fig. 3a). Without EGF treatment, cells harboring T790M and cells harboring L858R displayed higher levels of pAKT and pERK, as compared with other cells, indicating the constitutive phosphorylation in AKT and ERK by T790M and L858R (Fig. 3). With EGF treatment, pAKT was significantly increased in cells harboring T790M, L858R, WT, and some of HCC-derived mutants (K757E, N808S, and P937L), as compared to without EGF treatment (Fig. 3a, b). These three HCC-derived mutants (K757E, N808S, and P937L) were among the cells that displayed pEGFR at both Y869 and Y1092. With EGF treatment, pERK was significantly increased in all cells harboring EGFR WT and mutants (Fig. 3a, c). Notably, the increments of pAKT and pERK levels in cells harboring L858R treated with EGF were the least of all cells studied, and less than those of T790M and WT, indicating that the constitutive phosphorylation in AKT and ERK by L858R-mutant has almost reached its maximum by itself without EGF treatment. However, the pAKT and pERK levels in cells harboring HCC-derived EGFR mutants treated with EGF were much higher than their baseline without EGF, indicating that these HCC-derived EGFR mutants still response to EGF treatment and remain primarily EGF-dependent. Thus, these HCC-derived EGFR mutants are functioning, EGF-dependent, EGFRs which might affect their biological behavior.

### HCC-derived EGFR mutants induce increased cell proliferation higher than that of EGFR WT.

**Fig. 4:**
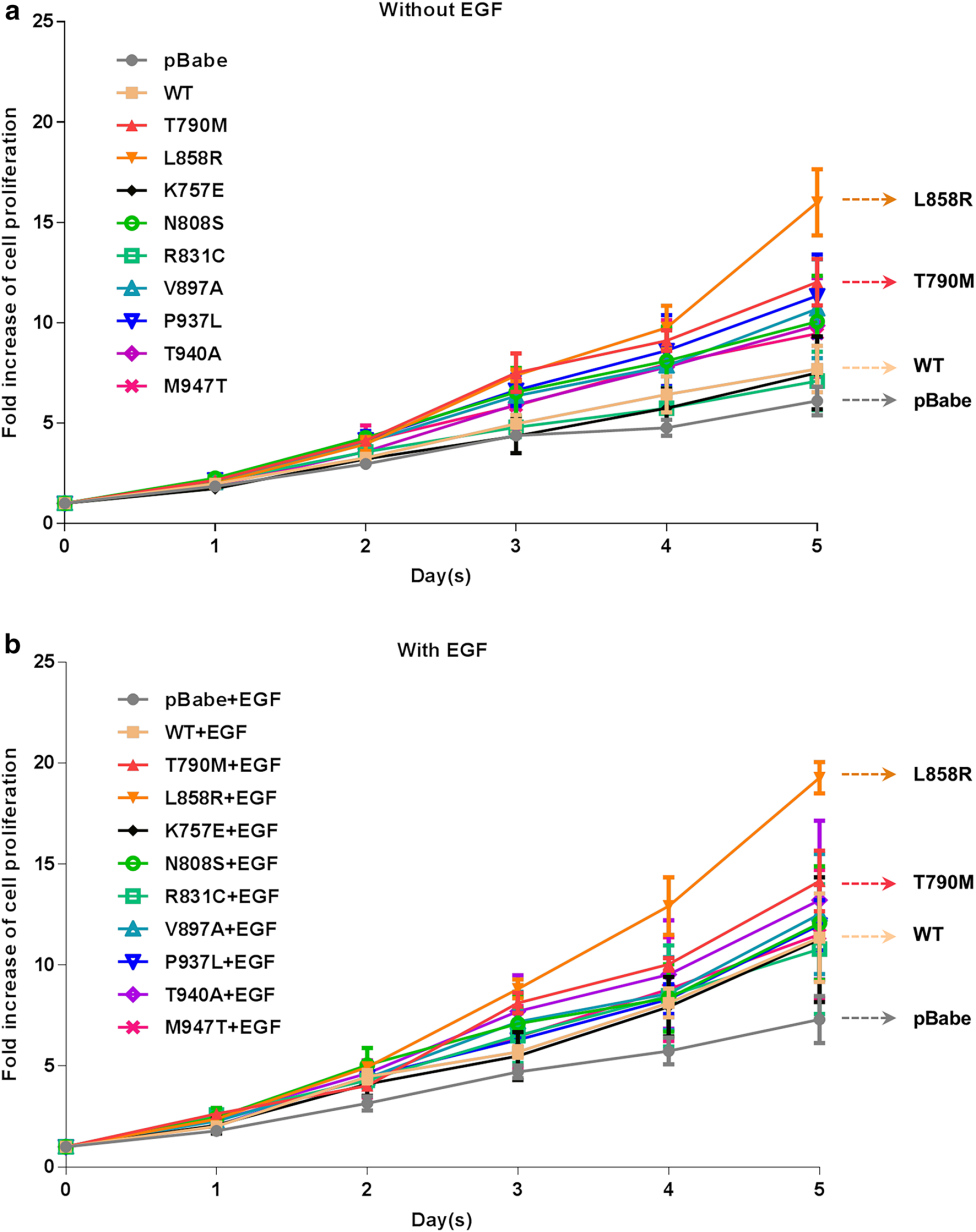
Growth curves of NIH-3T3 cells harboring EGFR WT and mutants treated with or without EGF. NIH-3T3 cells harboring EGFR WT and mutants with the same EGFR expression level were treated with DMEM containing 4% FBS without or with 50 ng/ml EGF (**a**, **b**, respectively). Cell viability was determined every day using MTT assay and growth curves plotted as fold increase of cell proliferation compared to day 0, presented as mean ± SEM

Proliferation of NIH-3T3 cells harboring vector alone (pBabe-puro) or EGFR WT and mutants was determined every day up to 5 days by MTT assay (Fig. 4) and the fold change of cell proliferation in day 5th of each clone was compared to day 0, as shown in Table 2. With or without EGF treatment, cells harboring vector alone displayed the lowest level of cell proliferation whereas cells harboring L858R and T790M displayed the highest level of cell proliferation, and some of the cells harboring HCC-derived EGFR mutants displayed increased cell proliferation higher than cells harboring vector alone and EGFR WT. Furthermore, all of the cells harboring EGFR WT and mutants showed additionally elevated cell proliferation when treated with EGF (Fig. 4a, b) (Table 2). These results indicate that the EGFR phosphorylation status, as well as the downstream AKT and ERK phosphorylation, in the absence of EGF is correlated with the growth of NIH-3T3 cells harboring both EGFR WT and mutants.

**Table 2:** The fold change of cell proliferation in day 5 of each EGFR mutation compared to day 0 and IC_50_ value of erlotinib treatment in each EGFR mutations

EGFR mutations	Fold change of cell proliferation		IC_50_ value (μM)
	Without EGF	With EGF	Erlotinib
pBabe	6.1 ± 0.7	7.3 ± 1.2	7.95 ± 0.4
WT	7.7 ± 1.2	11.4 ± 2.2	11.97 ± 1.8
T790M	12.0 ± 1.2	14.2 ± 1.5	15.16 ± 1.3
L858R	16.0 ± 1.6	19.3 ± 0.8	0.07 ± 0
K757E	7.5 ± 1.8	11.3 ± 3.1	24.67 ± 2.1
N808S	10.0 ± 2.3	12.1 ± 2.8	13.26 ± 5.3
R831C	7.1 ± 1.5	10.8 ± 3.2	8.47 ± 0.5
V897A	10.6 ± 2.5	12.5 ± 2.9	19.78 ± 3.3
P937L	11.3 ± 2.1	12.0 ± 1.2	12.32 ± 4.2
T940A	9.8 ± 2.3	13.2 ± 3.9	5.58 ± 0.4
M947T	9.5 ± 2.2	11.5 ± 3.2	12.3 ± 1.2

IC_50_ values for erlotinib in EGFR mutants were calculated by using the data from Fig. 3

### HCC-derived EGFR mutants remain erlotinib-resistant

**Fig. 5:**
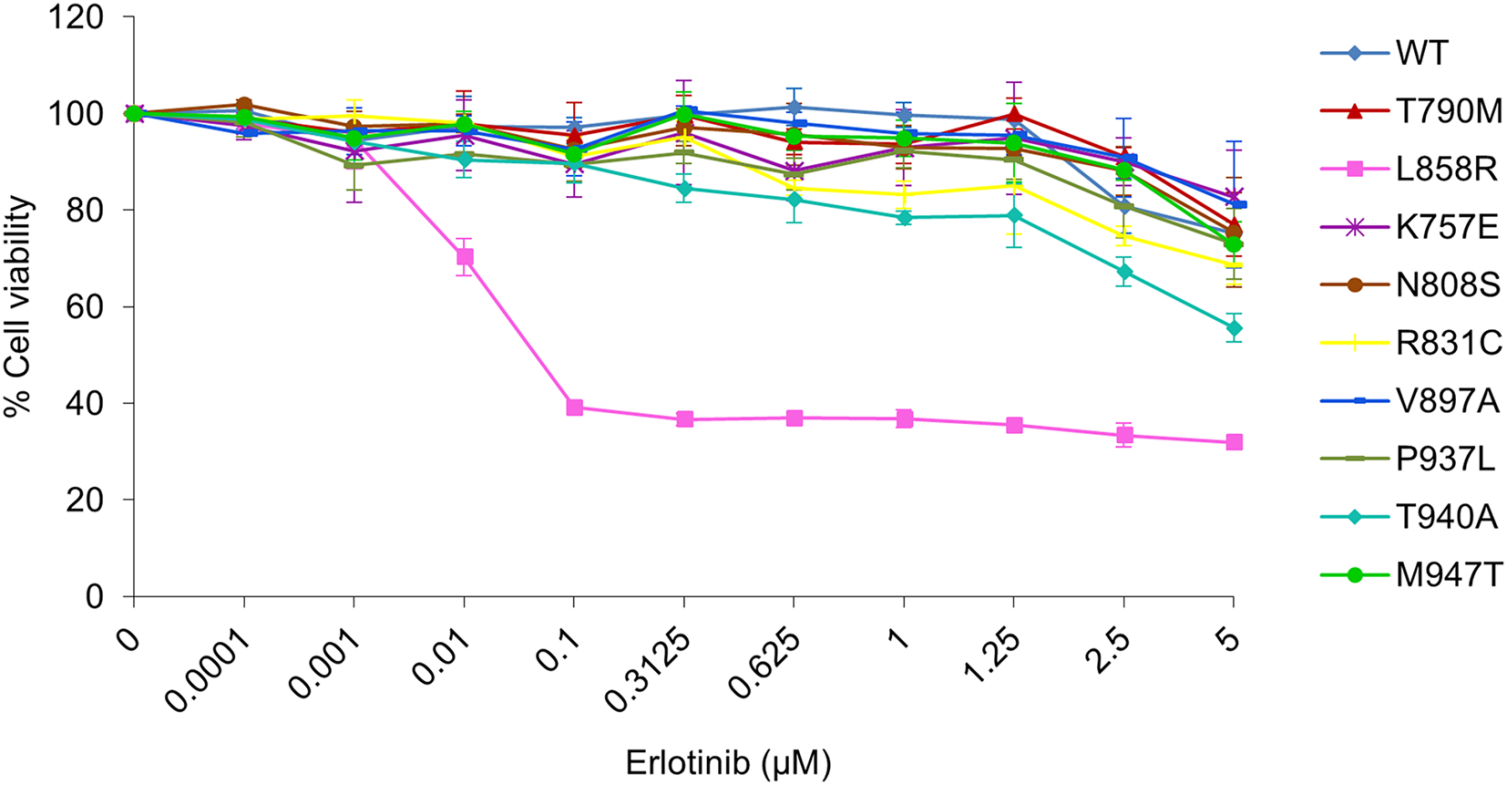
Cell viability curves of NIH-3T3 harboring EGFR WT and mutants treated with erlotinib. NIH-3T3 cells harboring EGFR WT and mutants were cultured in DMEM containing 4% FBS with erlotinib at various concentrations (0–5 µM) for 72 h. Cell viability was determined by MTT assay and cell viability curves were plotted as percentage of control untreated cells at the end of 72 h, presented as mean ± SEM

To determine the sensitivity to erlotinib, NIH-3T3 cells harboring EGFR WT and mutants were treated with erlotinib at various concentrations up to 5 µM for 72 h, followed by MTT assay (Fig. 5). Cells harboring T790M, the erlotinib-resistant control, and L858R, the erlotinib-sensitive control, showed the half-maximal inhibitory concentration (IC_50_) value of 15.16 ± 1.3 and 0.07 µM, respectively. Besides, all of the cells harboring HCC-derived mutants were suppressed only at the high concentration of erlotinib, namely the IC_50_ value of these cells being at more than 5 µM (Table 2). The partial response to the high concentration of erlotinib in cells harboring HCC-derived EGFR mutants indicates that they are erlotinib-resistant.

### In HCC-derived EGFR mutants, erlotinib could only induce partial inhibition of phosphorylation in EGFR, AKT and ERK

**Fig. 6:**
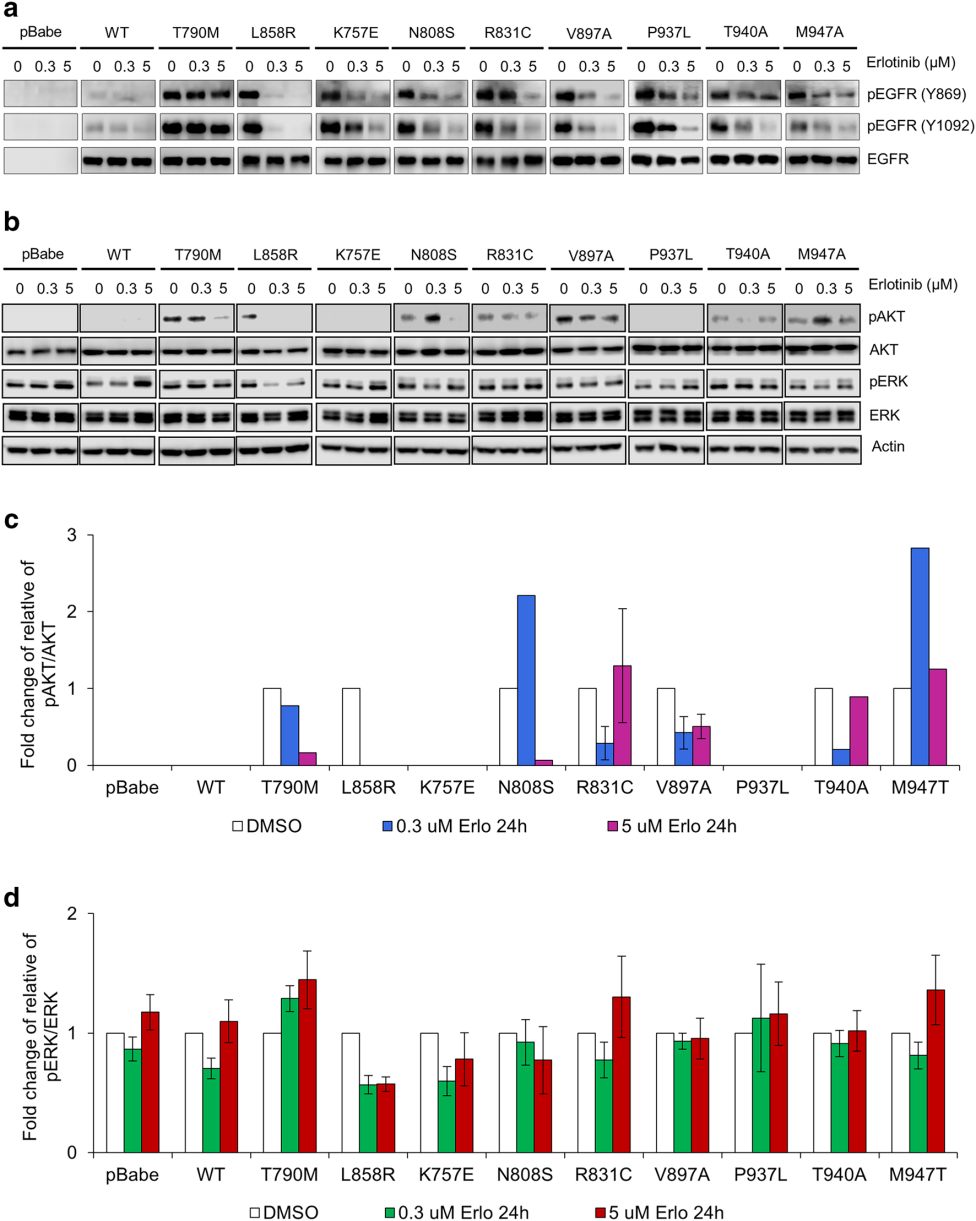
Erlotinib induces differential degree of inhibition of EGFR, Akt, and Erk phosphorylation among cells harboring different EGFRs. NIH-3T3 cells harboring EGFR WT and mutants were cultured in DMEM containing 4% FBS with different concentrations of erlotinib (0, 0.3, and 5 µM). Immunoblotting shows the effect of erlotinib on EGFR tyrosine phosphorylation after 24 h treatment (**a**), the effect of erlotinib on AKT and ERK phosphorylation after 24 h treatment, β-actin used as a loading control (**b**). Graphic presentations of relative band intensities of AKT and ERK phosphorylation in cells treated with erlotinib, presented as mean ± SEM in **c**, and **d** respectively

To determine the effect of erlotinib on phosphorylation in EGFR, AKT and ERK, NIH-3T3 cells harboring EGFR WT and mutants or vector alone were treated for 24 h with erlotinib at low (0.3 µM) and high concentration (5 µM). After erlotinib treatment, cells harboring T790M, the erlotinib-resistant control, did not show a reduced level of pEGFR on both Y869 and Y1092 at all, neither at low nor high concentration of erlotinib. In contrast, cells harboring L858R, the erlotinib-sensitive control, showed a remarkably reduced level of pEGFR on Y869 and Y1092 at low concentration of erlotinib and complete absence of pEGFR at high concentration of erlotinib (Fig. 6a). However, cells harboring EGFR WT and all HCC-derived EGFR mutants showed partially reduced levels of pEGFR on Y869 and Y1092 at high concentration of erlotinib.

After erlotinib treatment, cells harboring L858R, the erlotinib-sensitive control, showed a remarkably reduced level of pERK and complete absence of pAKT at low and high concentration of erlotinib (Fig. 6b–d). In contrast, cells harboring T790M, the erlotinib-resistant control, did not show a reduced level of pERK at all, neither at low nor high concentration of erlotinib, and showed a remarkably reduced level of pAKT only at high concentration of erlotinib (Fig. 6b–d). Besides, cells harboring EGFR WT and almost all HCC-derived EGFR mutants showed either minimal or no reduction in levels of pAKT and pERK at low and high concentration of erlotinib. Only one HCC-derived EGFR mutants (N808S) showed a remarkably reduced level of pAKT only at high concentration of erlotinib (Fig. 6b–d). The results confirm that cells harboring HCC-derived EGFR mutants are erlotinib-resistant, as treatment up to high concentration of erlotinib could only induce partial inhibition of EGFR phosphorylation, and partial or no inhibition of AKT and ERK phosphorylation.

### In HCC-derived EGFR mutants, erlotinib could only induce partial induction of apoptosis and autophagy

To determine the role of apoptosis and autophagy pathways in these erlotinib-resistant HCC-derived EGFR mutants, we used immunoblotting to monitor the effectors and molecular markers of apoptosis and autophagy: the activational cleavage of caspase-3, an executioner caspase; the cleavage of Poly (ADP-ribose) polymerase (PARP), the primary apoptotic substrate of active caspase-3 and caspase-7, and LC3-phosphatidylethanolamine conjugate (LC3-II), a surrogate marker of autophagy and autophagy-related processes [31, 32].

**Fig. 7:**
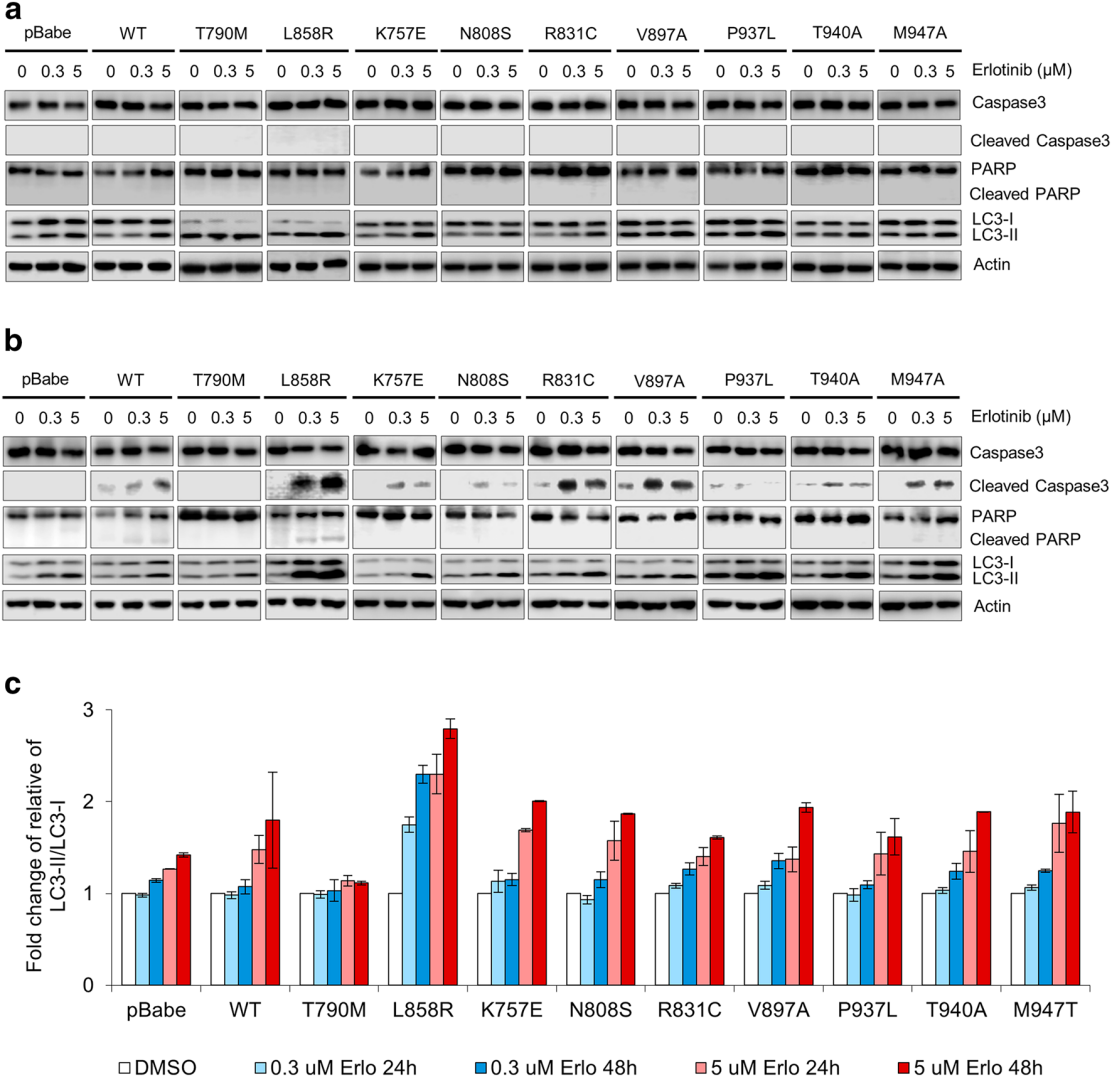
Erlotinib induces differential degree of induction apoptosis and autophagy among cells harboring different EGFRs. NIH-3T3 cells harboring EGFR WT and mutants were cultured in DMEM containing 4% FBS with different concentrations of erlotinib (0, 0.3, and 5 µM). Immunoblotting shows the effect of erlotinib on molecular markers of apoptosis and autophagy (caspase-3, PARP, LC3-I/II) after 24 h treatment (**a**) and 48 h treatment (**b**). Graphic presentations of the fold change of relative LC3-II/LC3-I in cells treated with erlotinib, presented as mean ± SEM (**c**)

After 24 h of erlotinib treatment, neither cleavage of caspase-3 nor cleavage of PARP was observed in NIH-3T3 cells harboring L858R, T790M, EGFR WT, and all HCC-derived EGFR mutants (Fig. 7a). However, after 48 h of erlotinib treatment, cleaved caspase-3 was clearly detectable in cells harboring L858R and also detectable at lesser extent in cells harboring EGFR WT and in most cells harboring HCC-derived EGFR mutants but still not detectable in cells harboring T790M (Fig. 7b). Nevertheless, cleaved PARP was detectable only in cells harboring L858R after 48 h of erlotinib treatment at both low and high concentration.

Regarding autophagy, the level of LC3-II was increased in dose- and time-dependent manner in cells harboring L858R treated with erlotinib; the increment was clearly detectable starting after 48 h of treatment with 0.3 µM erlotinib (Fig. 7b, c). In all cells harboring HCC-derived EGFR mutants, the LC3-II level was also clearly increased after 48 h of treatment with 5 µM erlotinib; the increment detected in cells harboring HCC-derived EGFR mutants was less and occurred later than in cells harboring L858R. In contrast, in cells harboring T790M, there was only minimal change of LC3-II level after treatment with 5 μM erlotinib up to 48 h; the change was the least of all groups (Fig. 7a–c).

All of these results indicate that erlotinib induces differential degree of apoptosis and autophagy among cells harboring different EGFRs: complete apoptosis and autophagy (cleavage of both caspase-3 and PARP, and marked LC3-II increment) in L858R-mutant; partial apoptosis and autophagy (only cleavage of caspase-3, and moderate LC3-II increment) in WT and HCC-derived mutants; and no apoptosis and minimal autophagy (no cleavage of caspase-3 and PARP, and minimal LC3-II increment) in T790M-mutant. The complete induction of both apoptosis and autophagy by erlotinib in cells harboring L858R-mutant is associated with severe inhibition of EGFR phosphorylation and consequently severe inhibition of AKT and ERK phosphorylation. No apoptosis and minimal autophagy in cell harboring T790M-mutant treated with erlotinib is associated with failure to inhibit EGFR and ERK phosphorylation, and with significant inhibition of AKT phosphorylation only at high concentration of erlotinib. The cells harboring HCC-derived EGFR mutants are erlotinib-resistant, as erlotinib up to high concentration could only induce partial inhibition of EGFR phosphorylation, and partial or no inhibition of AKT and ERK phosphorylation, and partial induction of apoptosis and autophagy.

## Discussion

In this study, we have shown that seven HCC-derived EGFR mutants (K757E, N808S, R831C, V897A, P937L, T940A, and M947T) identified by our previous study [24] are functioning, EGF-dependent, EGFRs; cells harboring six of the seven mutants could generate some level of EGFR tyrosine phosphorylation in the absence of EGF, indicating some constitutive kinase activity, but all of the seven mutants remain primarily EGF-dependent. Furthermore, these mutants are erlotinib-resistant, as treatment with erlotinib up to high concentration in cells harboring these mutants could only induce partial inhibition of EGFR phosphorylation, and partial or no inhibition of AKT and ERK phosphorylation, and partial induction of apoptosis and autophagy. Our results also indicate that erlotinib induce differential degree of apoptosis and autophagy among cells harboring different EGFRs. Besides, erlotinib-sensitivity, as determined by MTT assay, is correlated with a high degree of inhibition of EGFR, AKT and ERK phosphorylation, and with a high degree of erlotinib-induced apoptosis and autophagy.

In this study, it was found that even without EGF treatment, cells harboring T790M and L858R displayed a high level of EGFR, AKT and ERK phosphorylation, confirming their known constitutively active TKD. However, with EGF treatment, the increments of EGFR, AKT and ERK phosphorylation levels in cells harboring L858R were the least of all cells studied, and less than those of T790M and WT, indicating that the constitutive kinase activity of L858R-mutant has almost reached its full activity by itself without EGF treatment. This finding is consistent with the study by Shan et al. which uncovered the molecular mechanism of L858R mutation [33]. Using long-timescale molecular dynamics simulations, Shan et al. uncovered that the N-lobe dimerization interface of the wild-type EGFR kinase domain is intrinsically disordered and it becomes ordered only upon ligand-induced dimerization; besides, the common L858R mutation suppresses this intrinsic structural instability and promotes EGFR dimerization. Furthermore, L858R mutation causes abnormally high activity primarily by promoting EGFR dimerization rather than by allowing activation without dimerization [33]. As L858R mutation promotes EGFR kinase domain dimerization by itself, the effect of ligand-induced dimerization by EGF would be minimal, as shown in our finding. In contrast to cell harboring L858R, our results showed the significant additive effect of EGF on EGFR, AKT and ERK phosphorylation in cells harboring T790M, suggesting the difference between L858R and T790M mutation in catalytic activation, as indicated by the study of Sutto et al. [34]. Studying the conformational free-energy landscape of EGFR kinase among oncogenic mutations, Sutto et al. demonstrated that L858R mutation stabilizes the active conformation by a salt bridge between the positive charged R858 and the negatively charged glutamic acids 758 and 762 or the aspartic acid 761, thus strongly stabilizing the αC-helix and preventing the formation of inactive conformation. In contrast, T790M gatekeeper mutant favors activation by stabilizing a hydrophobic cluster around the phenylalanine F856 of the DFG motif in the activation loop [34].

Our results show that although cells harboring HCC-derived EGFR mutations could generate some basal EGFR tyrosine phosphorylation, indicating some constitutive kinase activity, they are erlotinib-resistant. Besides the EGFR-T790M mutation, the well-known 1^st^ generation EGFR TKI-resistant, EGFR-L861Q mutation also displays enhanced kinase activity and transforming potential, as compared with L858R and the wild type-EGFR kinase domain; however, L861Q does not increase drug sensitivity toward the 1^st^ generation EGFR TK (erlotinib, gefitinib) in contrast to the L858R [35]. Thus, the constitutive kinase activity of EGFR mutants is not the predictor of EGFR-TKI sensitivity.

Our results indicate that erlotinib induces differential degree of apoptosis and autophagy among cells harboring different EGFRs. In cells harboring drug-sensitive L858R mutant, our results have shown that the complete induction of both apoptosis and autophagy by erlotinib is associated with severe inhibition of EGFR phosphorylation and severe inhibition of the downstream AKT and ERK phosphorylation, which is consistent with previous studies demonstrating that EGFR TKIs trigger apoptosis via induction of the pro-apoptotic BH3-only BCL2 family member BIM [36–38], and also promote autophagy [39, 40]. In NSCLC cells harboring drug-sensitive EGFR mutants, EGFR TKI (erlotinib or gefitinib) triggers intrinsic (mitochondrial) apoptosis pathway via rapid increase in BIM levels and consequently the activation of the pro-apoptotic pore-former, BAX [36–38]. BIM status is regulated by the extracellular signal-regulated kinase (ERK) signaling cascade downstream of EGFR. Inhibition of ERK1/2 (extracellular signal-regulated protein kinase 1/2) signaling, but not inhibition of class I PI3K (phosphatidylinositol 3-kinase), JNK (c-Jun N-terminal kinase or mitogen-activated protein kinase 8), or AKT (protein kinase B), is essential for BIM activation [36–38].

Autophagy is an intracellular self-digestion process, by which cytosolic cellular components (“the cargo”) are sorted into the double-membrane autophagosomes and delivered to lysosomes for degradation [41]. Autophagy is initiated with the formation of a crescent-shaped phagophore (isolation membrane); this process is highly regulated by two critical kinases, the serine-threonine kinase ULK and the Class III phosphatidylinositol 3-kinase (Class III PI3K, also known as VPS34). ULK function requires a complex with FIP200 and ATG13, whereas VPS34 function requires a regulatory subunit, VPS15 (p150), and Beclin-1 [41]. Upon deprivation of growth factors or nutrients, inhibition of mTOR and/or activation of AMP result in activation of ULK, which then phosphorylates Beclin-1 on serine residues, and consequently promoting the formation of the active Beclin 1-VPS34 complex [42]. Then, the active Beclin 1/VPS34 complex generates phosphatidylinositol 3, 4, 5-triphosphate (PI3P) on the membrane inevitable to become a phagophore, and PI3P recruits proteins required for phagophore elongation. Phagophore elongation requires the incorporation of LC3-phosphatidylethanolamine conjugate (LC3-II) [43]. The phagophore elongates until its membranes fuse, generating a mature autophagosome [41].

EGFR signaling activates the class I PI3K-AKT-mTORC1 pathway, which is known to negatively regulate autophagy [44]. Hence, a study in NSCLC cell lines harboring EGFR wild type has shown that gefitinib promotes autophagy and apoptosis, leading to lung cancer cell death, via blockade of the PI3K/AKT/mTOR pathway [40]. Recently, Wei et al. uncovered another novel mechanism of EGFR suppression of autophagy; this mechanism is mTOR-independent and it involves an interaction between EGFR and the Beclin 1 autophagy protein [39]. Both ligand-dependent activation of wild type EGFR and activating mutations in EGFR TKD (notably L858R-mutant and Exon19 del746–750) lead to the EGFR endocytosis and the formation of an EGFR/Beclin 1 complex residing primarily in endosomes. Consequently, the activated EGFR mediates Beclin 1 tyrosine phosphorylation, which promotes Beclin1 homodimerization and enhances the interaction of Beclin 1 with negative regulators such as Bcl-2 and Rubicon, and disrupts the interaction of Beclin 1 with VPS34, resulting in suppression of autophagy. Erlotinib, an ATP-competitive inhibitor of EGFR, dephosphorylates EGFR and abolishes the EGFR/Beclin 1 interaction, leading to autophagy in TKI-sensitive, but not TKI-resistant cells. TKI-induced autophagy in NSCLCs with active EGFR is associated with increased Beclin 1-associated VPS34 kinase activity and disruption of the interaction between Beclin 1 and EGFR and between Beclin 1 and negative regulators of autophagy such as Bcl-2 and Rubicon [39].

In summary, we have shown that (1) seven of HCC-derived EGFR mutants (K757E, N808S, R831C, V897A, P937L, T940A, and M947T) identified by our previous study [24] are functioning, EGF-dependent, and erlotinib-resistant; (2) erlotinib induces differential degree of apoptosis and autophagy among cells harboring different EGFRs mutants; (3) the degree of inhibition of EGFR phosphorylation by erlotinib is the determining factor for the degree of apoptosis and autophagy amongst cells harboring EGFR mutants. This study paves the way for further investigation into the sensitivity of these HCC-derived mutants to the 3rd-generation irreversible EGFR TKI, osimertinib.

## Supplementary information

**Additional file 1: Figure S1.** The confirmation of DNA sequences of EGFR mutation. The DNA sequences of each EGFR mutations after site-directed mutagenesis in pBabe-puro were confirmed with sequencing by Macrogen. Inc., South Korea.

## Data Availability

The datasets supporting the conclusions of this article are included within the article.
