# Supplementary figures and images for: HCC-derived EGFR mutants are functioning, EGF-dependent, and erlotinib-resistant
Source: Cell Biosci. 2020 Mar 16;10:41. doi: 10.1186/s13578-020-00407-1 (PMC7076995; doi:10.1186/s13578-020-00407-1)

## Slide 1
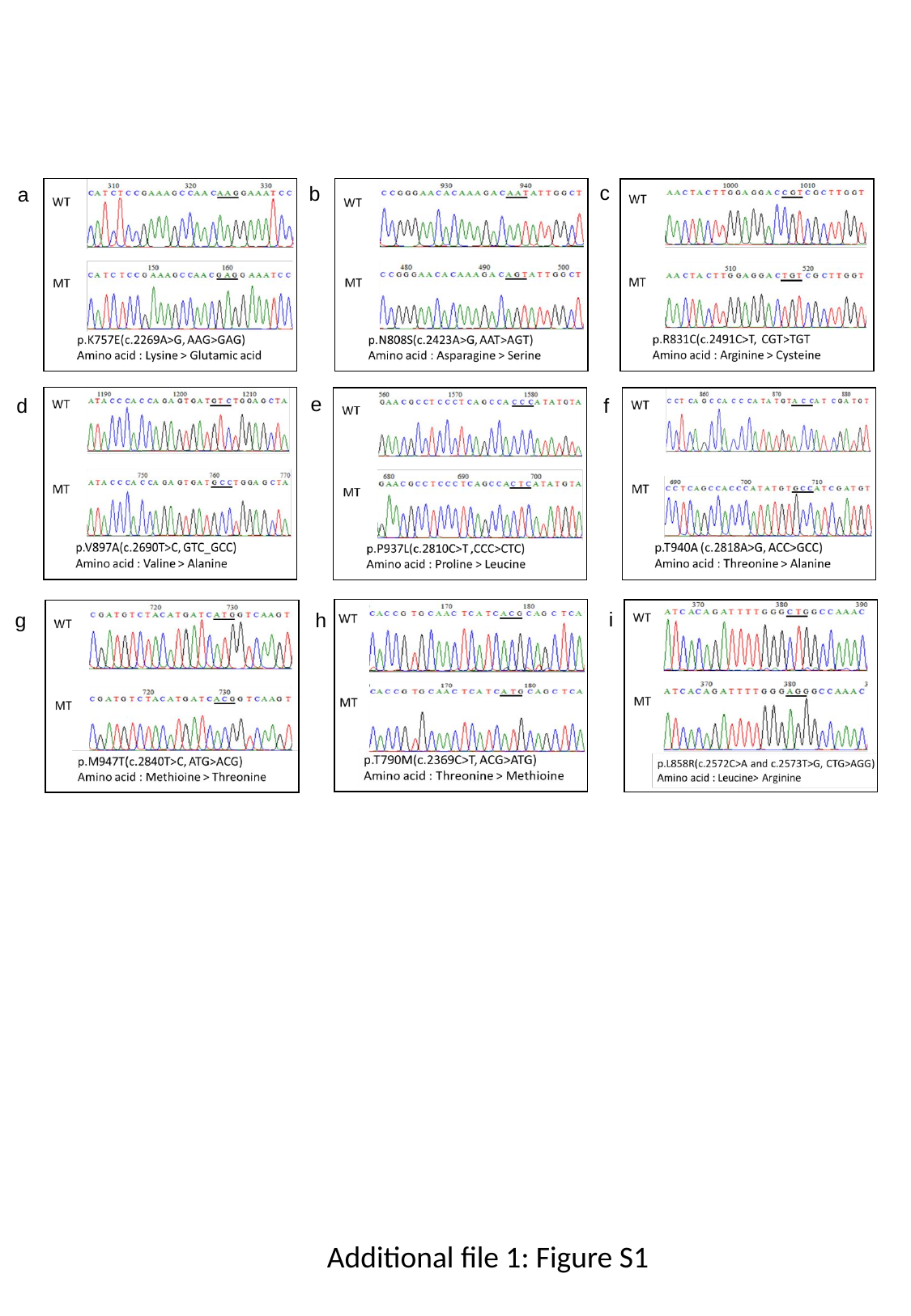

c
b
a
e
f
d
i
h
g
Additional file 1: Figure S1

Supplement: Supplementary file 1 — Additional file 1: Figure S1. The confirmation of DNA sequences of EGFR mutation. The DNA sequences of each EGFR mutations after site-directed mutagenesis in pBabe-puro were confirmed with sequencing by Macrogen. Inc., South Korea. [file 13578_2020_407_MOESM1_ESM.pptx]
